# The evaluation of a remote support program on quality of life and evolution of disease in COPD patients with frequent exacerbations

**DOI:** 10.1186/s12890-016-0304-3

**Published:** 2016-11-08

**Authors:** Bernardino Alcazar, Pilar de Lucas, Joan B. Soriano, Alonso Fernández-Nistal, Antonia Fuster, Jose Miguel Rodríguez González-Moro, Aurelio Arnedillo, Patricia García Sidro, María José Espinosa de los Monteros

**Affiliations:** 1 Hospital de Alta Resolución de Loja, Agencia Sanitaria H. de Poniente, Avda Tierno Galván s/n., CP 18300 Loja, Granada Spain; 2 Hospital Universitario Gregorio Marañón, Madrid, Spain; 3 Instituto de Investigación Hospital Universitario de la Princesa (IISP), Universidad Autónoma de Madrid, Madrid, Spain; 4 Departamento médico Takeda Farmacéutica España S.A., Madrid, Spain; 5 Hospital Son Llàtzer, Mallorca, Spain; 6 Hospital Universitario Puerta del Mar, Cádiz, Spain; 7 Hospital de la Plana, Castellón, Spain; 8 Hospital Universitario Virgen de la Salud, Toledo, Spain

**Keywords:** Chronic obstructive pulmonary disease, Education, Management, Quality of life, Exacerbations

## Abstract

**Background:**

Chronic obstructive pulmonary disease (COPD) patients often present considerable individual medical burden in their symptoms, limitations, and well-being that complicate medical treatment. To improve their overall health status, while reducing the number of exacerbations, a multidisciplinary approach including different elements of care is needed. The aim of this study was to evaluate the effects of a remote support program on COPD patients at high risk of experiencing worsening of their disease and other health-related outcomes.

**Methods:**

An observational, multicenter, prospective study aimed at evaluating the impact of a 7-month remote support program on COPD patients in exacerbations control and changes in health status measured with the COPD assessment test (CAT). Factors associated with a clinically relevant decrease in CAT were assessed using a logistic regression analysis.

**Results:**

A total of 114 subjects started the program. The majority of the study population were males (81.6 %), retired (70.2 %), without academic qualifications or with a low level of education (68.4 %), and ex-smokers (79.8 %). The mean ± SD age was 69.6 ± 9.1 years and the BMI was 27.8 ± 5.5 Kg/m^2^. Overall, 41.9 % (95 % CI 31.9–52.0) patients, significantly improved health status (CAT decrease ≥ 2 points). Univariate analysis showed that significant improvement in CAT was associated with baseline CAT scores [high CAT score 19.2 (±7.5) vs. low CAT score 12.4 (±6.4); OR = 1.15, 95 % CI: 1.07–1.24; *p* < 0.001] and with being non-compliant [62.5 % (15/24) of non-compliant vs 34.7 % (24/69) of compliant patients significantly improved CAT scores; OR = 3.13, 95 % CI: 1.19–8.19; *p* = 0.021). After controlling for the effect of all variables in a multivariable logistic regression model, the only factor that remained significant was baseline CAT score. The proportion of smokers in the total population remained constant during the study. There was a significant reduction in the number of exacerbations after entering this remote support program with median -1 (IQR: -2, 0), (*p* < 0.001). The Morisky-Green questionnaire showed an increase of treatment compliance, namely at baseline, 25.8 % (24/93) of patients were noncompliant while in the end 66.7 % (16/24) of them became compliant) (*p* = 0.053).

**Conclusions:**

A remote support program for high-risk COPD patients results in an improvement of the patients’ health status, particularly in those with initially poor health status, and it helps to reduce COPD exacerbations.

**Electronic supplementary material:**

The online version of this article (doi:10.1186/s12890-016-0304-3) contains supplementary material, which is available to authorized users.

## Background

Chronic obstructive pulmonary disease (COPD) is considered a preventable, treatable, disabling respiratory disease characterized by an often progressive and mainly irreversible airflow obstruction [1, 2]. COPD is currently the fourth leading cause of death worldwide, and is projected to be the third cause of death in developed countries by 2020 [3, 4].

COPD is a paradigm of chronic disease, in which self-management and support are essential for a proper control [5]. Primary medical management of COPD often focuses on improving airflow using bronchodilators and anti-inflammatory therapies; however, the airflow obstruction associated with COPD is not completely reversible and often tends to progressively worsen over time [6]. Much of the deterioration and progression of the disease is related to the number and severity of exacerbations experienced by patients [7] and, as the disease progresses, the patients experience a worsening in their quality of life [8]. Because complete recovery/cure from COPD is impossible, health professionals should focus on the improvement of patient-centered outcomes including health status and quality of life; which are important outcome measures for treatment and care in COPD patients [9].

Disease-specific programs are an integral component of collaborative self-management. This approach is recognized to improve health outcomes in people with chronic conditions [10] and has also successfully improved the health related quality of life (HRQoL) of patients with chronic respiratory disease, such as asthma [11]. These programs provide information for patients to recognize and therefore to prevent, and decrease the severity and/or frequency of symptoms and to implement appropriate treatment for the episodes [11].

A number of studies have evaluated the effect of different education programs for COPD and have concluded that participation in these programs was associated with an increase in the knowledge of COPD, an improvement in specific skills to manage the disease, an increased adherence to inhaled therapy, and a decrease in emergency room visits and hospital admissions due to COPD exacerbations; among other positive outcomes [12–15]. However, the available evidence is still inconclusive due to different study designs, different types of COPD patients included, methodological limitations, and a wide variation of reported outcome measures.

We hypothesized that the implementation in a real clinical practice setting of a disease-specific remote support program (Horizonte program) for COPD patients at high risk, would positively influence these patients’ health status. The Horizonte program basically consists of sending text messages, e-mails, and conducting regular calls by qualified nurses, to facilitate proper monitoring of the disease (www.atlantishealthcare.com). It has been previously used in Spain under controlled conditions, to assure its applicability in COPD patients, but has not been incorporated in to common clinical practice until just recently.

Thus, the primary aim of the study was to assess the outcomes of the Horizonte remote support program for COPD patients at high risk, in terms of reduction in frequency and severity of exacerbations, and improvement in the patients’ health status measured with the COPD assessment test (CAT) when used as a part of the usual treatment.

## Methods

### Design of the study and data collection

This was an observational, multicenter, prospective study where 24 pulmonologists, distributed throughout the Spanish national territory centres, recruited consecutive patients between November 2013 and October 2014. Each researcher aimed to recruit 9 consecutive patients.

### Study population

Patients of both genders, aged 40 years or older, fulfilling the selection criteria who signed the informed consent were recruited. The inclusion criteria were: a) COPD confirmed by spirometry performed in stable state not more than 12 months prior to recruitment in the study with a post-bronchodilator ratio of FEV1/FVC < 0.7; b) smoker or former smoker of at least 10 pack-years; c) patients with 2 or more moderate/severe exacerbations reported in the 12 months prior to study entry; d) patient clinically stable at the time of inclusion in the study and who was willing to participate in the Horizonte patient support program.

The exclusion criteria in the study were: (i) patients who had never smoked, (ii) those who suffered a moderate/severe exacerbation in the previous 30 days (iii) other chronic respiratory disease (e.g. bronchial asthma, allergic rhinitis, severe bronchiectasis, cancer, restrictive lung disease, etc.) or pulmonary surgery, (iv) or who, in the opinion of the investigator, did not demonstrate sufficient cognitive capacity; presented sensory or psychiatric disability or language barriers that might prevent or hinder participation in the study, (v) and participation in another study or clinical trial.

An observation period of 12 months that included 7 months of allocation in the Horizonte remote support program and a follow-up period of 5 months was established. An evaluation was carried out before and after a 2 and 5-month period posterior to the inclusion in the study, in which the effectiveness of the measures implemented in the support program were assessed. Each patient conducted their own self-monitoring, comparing their health status sequentially. For each patient included, data was collected in an electronic data collection notebook (e-CDR) designed for this purpose. The information requested in this e-CDR conformed to usual clinical practice, and referred to in the standard management of patients with COPD.

### Horizonte program

The Horizonte program (developed by the company Atlantis Healthcare) (15) is based on the sending of text messages (SMS) and e-mails, in addition to calls made by skilled nurses, to patients who have given their consent. The program has been endorsed by the Spanish Pulmonology and Thoracic Surgery Society (SEPAR) and it is a support program for patients with COPD and was designed to help them understand their disease and treatment by changing their misconceptions, improving adherence to prescribed treatments and healthy habits (eg. reducing smoking), and consequently their quality of life. The program is available for both newly diagnosed COPD patients and those already diagnosed and treated. Initially, the risk of non-adherence is assessed in each patient by the completion of a questionnaire. According to these results, segments and risk levels are set (high or low risk of non-adherence), and different interventions are established: Telephone calls from nurses, notebooks in which the objectives are preset to guide the patient, magazines (5 issues for high risk and 2 issues for low risk), additional documentation focused on the specific problems of each patient (max. 4 per patient), and SMS and e-mails whose frequency varies depending on the patient’s risk (see a complete description at the end of the manuscript).

The program’s objective is to improve patient education with regard to the knowledge of their disease and to achieve better adherence. Patient coordination is led by the company Atlantis Healthcare and is funded by Takeda Pharmaceutical Spain SA. A more detailed description of the Horizonte program can be reviewed at Additional file 1.

### Study variables

Recruiting physicians collected information regarding demographic data, smoking, medical history, comorbidities, physical examination (weight, height, BMI, abdominal circumference), pharmacological treatment, history of exacerbations in the past 7 months, number of hospitalizations for COPD in the past 7 months, spirometry (lung function), Morisky-Green and Levine Tests, Battle test, exacerbations from baseline, and hospitalizations. The cardiovascular risk was assessed according to BMI, gender, and waist circumference [16].

Patients were asked to fill in the CAT questionnaire at baseline (VB) and after 2 (V2), 7 (last Horizonte program visit – V3) and 12 months (FV) in its validated Spanish version. The CAT consists of 8 items with scores ranging from 0 to 5 (0 = no impairment). An overall score is calculated by adding the score from each item with total scores ranging from 0 to 40; a higher score indicates a more severe health status impairment or a poorer control of COPD [17, 18]. The CAT’s minimal clinical important difference (MCID) has not yet been established, and has been estimated to be 2.0 or more points [19].

“Moderate COPD exacerbations were defined as a sudden increase in respiratory symptoms that required ambulatory treatment with systemic corticosteroids and/or antibiotics, and exacerbations were considered severe when the patient required hospitalization”.

### Sample size calculation

For the sample size calculation, it was estimated that 171 completers were needed to detect a mean change in CAT of at least 2.5 (±10.0) points, with 90 % power and an alpha of 0.05. Assuming a dropout rate of 20 % from the start of the study, the number of patients recruited should total 214.

### Statistical analysis

To describe the qualitative variables, absolute frequencies and percentages were used. For quantitative variables, those normally distributed used mean, standard deviation (SD), minimum and maximum figures, while median, interquartile range, minimum and maximum were used when they were not normally distributed.

Variables of interest were compared between study groups using the Chi-squared or the Fisher’s exact test for categorical variables, and the Student’s “t” test for independent data (or the Mann-Whitney *U* test if the assumption of normality was not met), to compare quantitative variables. The Kolmogorov-Smirnov test or Shapiro-Wilk test were used to assess if the quantitative variables followed a normal distribution. Comparisons of quantitative variables between more than two groups were made using the Kruskal-Wallis test or analysis of variance (ANOVA), depending on data distribution.

The pre and post-intervention comparisons of quantitative variables were performed using the t Student test or the Wilcoxon test, depending on the data distribution. The pre and post-intervention comparisons of qualitative variables of two categories were performed using the McNemar test.

Incidence rates of exacerbations were described by annualized incidence rates with their 95 % confidence interval (95 % CI). Annualization of the rates and their corresponding confidence intervals were performed by adjusting to a Poisson model incorporating the time (expressed in years) of each patient in the study as incidence rates with their corresponding 95 % CI, and *p*-values. The analysis of a clinically significant change in CAT was assessed by logistic regression analysis, and expressed by the odds ratio (OR) with its 95 % confidence interval and *p* values. Changes in CAT scores throughout the study were analyzed using a generalized linear mixed model of repeated measures. Data analysis was performed using the Statistical package SAS version 9.4 and statistical significance was considered when *p* < 0.05.

## Results

### Subject characteristics

**Fig. 1:**
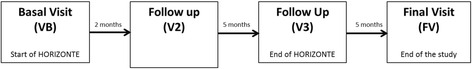
Flow Diagram of the study

**Fig. 2:**
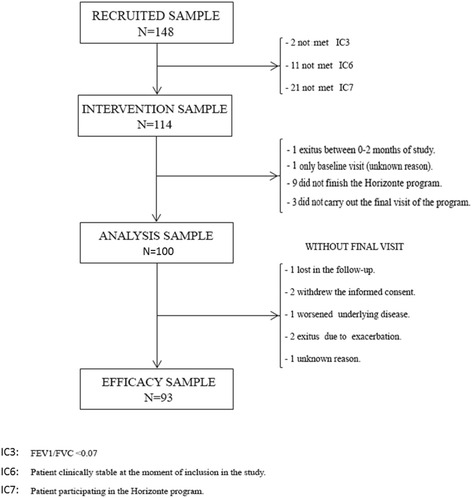
Flow-chart of the study

The study flow-diagram and flow-chart are shown in Figs. 1 and 2. A total of 148 subjects were initially recruited in the study. Thirty-four were excluded because they did not meet all the inclusion criteria (*n* = 114), then 100 completed the intervention program, and finally 93 completed all study procedures (62.8 %). Baseline demographic characteristics of the patients are shown in Table 1. Most participants were male (81.6 %) and were ex-smokers (79.8). Mean age was 69.6 years ± 9.1 and mean BMI was 27.8 ± 5.5 Kg/m^2^. Clinical characteristics and pulmonary function parameters of the sample at baseline are shown in Tables 2 and 3, respectively. A total of 90 subjects (78.9 %) had been vaccinated against influenza. Mean FVC and FEV1 (%) at the baseline were 71.3 % (±19.4) and 48.7 % (17.4 %) %, respectively. Regarding COPD severity by spirometric GOLD stage, 5.3 % had mild COPD, 36.8 % moderate, 43.9 % severe and 14.0 % very severe COPD. At baseline, 98.2 % (112/114) of participating patients were receiving treatment/s for COPD (Tables 1, 2 and 3)

**Table 1:** Socio-demographic characteristics of the study population

		Overall	Treatment non-adherence based on Morisky-Green baseline score		
			High-Risk	Low-Risk	*p*-value
		*N* = 114	27 (23.7)	87 (76.3)	
Gender					
Men	n(%)	93 (81.6)	18 (66.7)	75 (86.2)	0.043^F^
Women	n(%)	21 (18.4)	9 (33.3)	12 (13.8)	
Missing	n	0	0	0	
Age (years)					
Mean (S.D.)		69.6 (9.1)	68.3 (10.0)	70.0 (8.8)	0.449^U^
≤ 65 years	n(%)	44 (38.6)	11 (40.7)	33 (37.9)	0.793^C^
> 65 years	n(%)	70 (61.4)	16 (59.3)	54 (62.1)	
Missing	n	0	0	0	
Level of education					
No studies/Primary studies	n(%)	78 (68.4)	17 (63.0)	61 (70.1)	0.485^C^
Intermediate and higher education	n(%)	36 (31.6)	10 (37.0)	26 (29.9)	
Missing	n	0	0	0	
Employment situation					
Retired	n(%)	80 (70.2)	16 (59.3)	64 (73.6)	0.156^C^
Other	n(%)	34 (29.8)	11 (40.7)	23 (26.4)	
Missing	n	0	0	0	
BMI (kg/m^2^)					
Mean (S.D.)		27.8 (5.5)	27.8 (6.2)	27.8 (5.3)	0.931^U^
Underweight (<18.5)	n(%)	3 (2.6)	1 (3.7)	2 (2.3)	0.738^F^
Normal weight (18.5–24.9)	n(%)	36 (31.6)	10 (37.0)	26 (29.9)	
Overweight (25.0–29.9)	n(%)	41 (36.0)	8 (29.6)	33 (37.9)	
Obesity (≥30.0)	n(%)	34 (29.8)	8 (29.6)	26 (29.9)	
Missing	n	0	0	0	
Cardiovascular risk					
None	n(%)	31 (27.2)	8 (29.6)	23 (26.4)	0.907^C^
Increased	n(%)	27 (23.7)	5 (18.5)	22 (25.3)	
High	n(%)	29 (25.4)	7 (25.9)	22 (25.3)	
Very/Extremely high	n(%)	27 (23.7)	7 (25.9)	20 (23.0)	
Missing	n	0	0	0	
Smoking status					
Ex-smoker	n(%)	91 (79.8)	17 (63.0)	74 (86.1)	0.012^C^
Smoker	n(%)	23 (20.2)	10 (37.0)	13 (14.9)	
Missing	n	0	0	0	
COPD treatment (at baseline)	n(%)	112 (98.2)	27 (100.0)	85 (97.7)	1.000^C^
Long-acting B2 agonists	n(%)	101 (90.2)	23 (85.2)	78 (91.8)	0.456^F^
Long-acting anticholinergics	n(%)	99 (88.4)	24 (88.9)	75 (88.2)	1.000^F^
Phosphodiesterase-4 Inhibitors	n(%)	30 (26.8)	6 (22.2)	24 (28.2)	0.539^C^
Inhaled Corticosteroids	n(%)	88 (78.6)	17 (63.0)	71 (83.5)	0.023^C^
Oxygen therapy	n(%)	32 (28.6)	4 (14.8)	28 (32.9)	0.069^C^
non-invasive mechanical ventilation	n(%)	12 (10.7)	4 (14.8)	8 (9.4)	0.479^F^
Missing	n	0	0	0	

*COPD* chronic obstructive pulmonary disease, *S.D* standard deviation, *BMI* body mass index

^C^Chi-square test; ^F^Fisher’s exact test; ^U^Mann-Whitney *U* test

**Table 2:** Clinical characteristics

		Overall	Treatment non-adherence based on Morisky-Green baseline score		
			High-Risk	Low-Risk	*p*-value
		*N* = 114	27 (23.7)	87 (76.3)	
Time of evolution of COPD (years)					
Mean (S.D.)		8.8 (6.8)	7.7 (6.2)	9.2 (7.0)	0.314^U^
Missing		1	0	1	
Number of moderate exacerbations in the last 12 months					
Mean (S.D.)		1.6 (1.1)	1.5 (0.8)	1.6 (1.2)	0.967^U^
Missing		0	0	0	
Number of severe exacerbations in the last 12 months					
Mean (S.D.)		1.2 (1.6)	0.7 (1.0)	1.4 (1.8)	0.049^U^
Missing		0	0	0	
Total number of exacerbations in the last 12 months					
Mean (S.D.)		2.8 (1.4)	2.2 (0.5)	3.0 (1.5)	0.003^U^
2–3 exacerbations	n(%)	97 (85.1)	26 (96.3)	71 (81.6)	0.069^F^
4 or more exacerbations	n(%)	17 (14.9)	1 (3.7)	16 (18.4)	
Missing		0	0	0	
Comorbidities					
Cardiac comorbidities	n(%)	41 (36.0)	8 (29.6)	33 (37.9)	0.432^C^
Coronary heart disease (CHD)	n(%)	22 (19.3)	4 (14.8)	18 (20.7)	0.499^C^
Peripheral vascular disease	n(%)	13 (11.4)	2 (7.4)	11 (12.6)	0.730^F^
Missing	n	0	0	0	

*COPD* chronic obstructive pulmonary disease, *S.D* standard deviation

^C^Chi-square test; ^F^Fisher’s exact test; ^U^Mann-Whitney *U* test

**Table 3:** Lung function at baseline

		Overall	Treatment non-adherence based on Morisky-Green baseline score		
			High-Risk	Low-Risk	*p*-value
		*N* = 114	27 (23.7)	87 (76.3)	
Spirometry					
No	n(%)	0 (0.0)	-----	-----	
Yes, prior to the visit	n(%)	75 (65.8)	13 (48.2)	62 (71.3)	0.027^C^
Yes, during the visit	n(%)	39 (34.2)	14 (51.8)	25 (28.7)	
Spirometry results					
FVC (ml) - Mean (S.D.)		2485.4 (844.0)	2546.7 (788.7)	2466.4 (864)	0.668^T^
FVC (%) - Mean (S.D.)		71.3 (19.4)	75.7 (21.7)	69.9 (18.6)	0.142^U^
FEV1 (ml) - Mean (S.D.)		1239.7 (521.8)	1456.3 (544.9)	1172.5 (498.7)	0.013^T^
FEV1 (%) - Mean (S.D.)		48.7 (17.4)	56.7 (17.8)	46.2 (16.6)	0.007^U^
FEV1/FVC (%) - Mean (S.D.)		50.8 (13.2)	57.3 (10)	48.8 (13.5)	0.004^U^
Missing	n	0	0	0	
GOLD stage					
Stage I: Mild (80 ≤ FEV1% ≤ 100)	n(%)	6 (5.3)	2 (7.4)	4 (4.6)	0.074^F^
Stage II: Moderate (50 ≤ FEV1% ≤ 79)	n(%)	42 (36.8)	15 (55.6)	27 (31.0)	
Stage III: Severe (30 ≤ FEV1% ≤ 49)	n(%)	50 (43.9)	7 (25.9)	43 (49.4)	
Stage IV: Very severe (FEV1% < 30)	n(%)	16 (14.0)	3 (11.1)	13 (14.9)	
Missing	n	0	0	0	
O_2_ saturation (%)					
X (D.E.)		93.9 (3.0)	95.0 (2.7)	93.5 (3.0)	0.025^U^
O_2_ saturation >90 %		98 (86.0)	25 (92.6)	73 (83.9)	0.351^F^
Missing	n	0	0	0	
Minutes of walking per day					
< 30	n(%)	39 (34.2)	6 (22.2)	33 (37.9)	0.323^C^
30–60	n(%)	36 (31.6)	10 (37.0)	26 (29.9)	
> 60	n(%)	39 (34.2)	11 (40.7)	28 (32.2)	
Missing	n	0	0	0	
Emphysema					
No	n(%)	56 (49.1)	18 (66.7)	38 (43.7)	0.037^C^
Yes	n(%)	58 (50.9)	9 (33.3)	49 (56.3)	
Missing	n	0	0	0	
BODEx index					
Quartile 1: 0–2 points	n(%)	28 (24.6)	12 (44.4)	16 (18.4)	0.011^C^
Quartile 2: 3–4 points	n(%)	44 (38.6)	11 (40.7)	33 (37.9)	
Quartile 3: 5–6 points	n(%)	31 (27.2)	4 (14.8)	27 (31.0)	
Quartile 4: 7–10 points	n(%)	11 (9.6)	0 (0.0)	11 (12.6)	
Missing	n	0	0	0	

*S.D* standard deviation

^C^Chi-square test; ^F^Fisher’s exact test; ^T^ *T*-Test; ^U^Mann-Whitney *U* test

### Changes in smoking status and pharmacological treatment

During the study, most participants (93.6 %) did not change their smoking status. However, from baseline up to 2 months prior to inclusion (V2), 4.3 % (4/93) of participants changed their smoking status (2 gave up smoking, one significantly decreased the number of cigarettes/day, and another significantly increased the number of cigarettes/daily). Between V2 and V3, 4.3 % (4/93) of patients changed their smoking status, (2 gave up smoking, one resumed smoking again, and one significantly reduced the number of cigarettes/day). Finally, between the V3 and FV, 2.2 % (2/93) of patients significantly reduced the number of cigarettes/day.

During the baseline visit, COPD treatment was changed in 36.0 % (41/114) of patients. At the first follow-up visit (V2), 99.1 % (111/112) of patients were receiving treatment for COPD and during the visit; the treatment was changed in 25.9 % (29/112) of them. At the last visit of the program (V3) and at the end of the study (FV), all patients were receiving treatment for COPD. Changes in adherence are stated at the end of this section.

### Changes in CAT

**Fig. 3:**
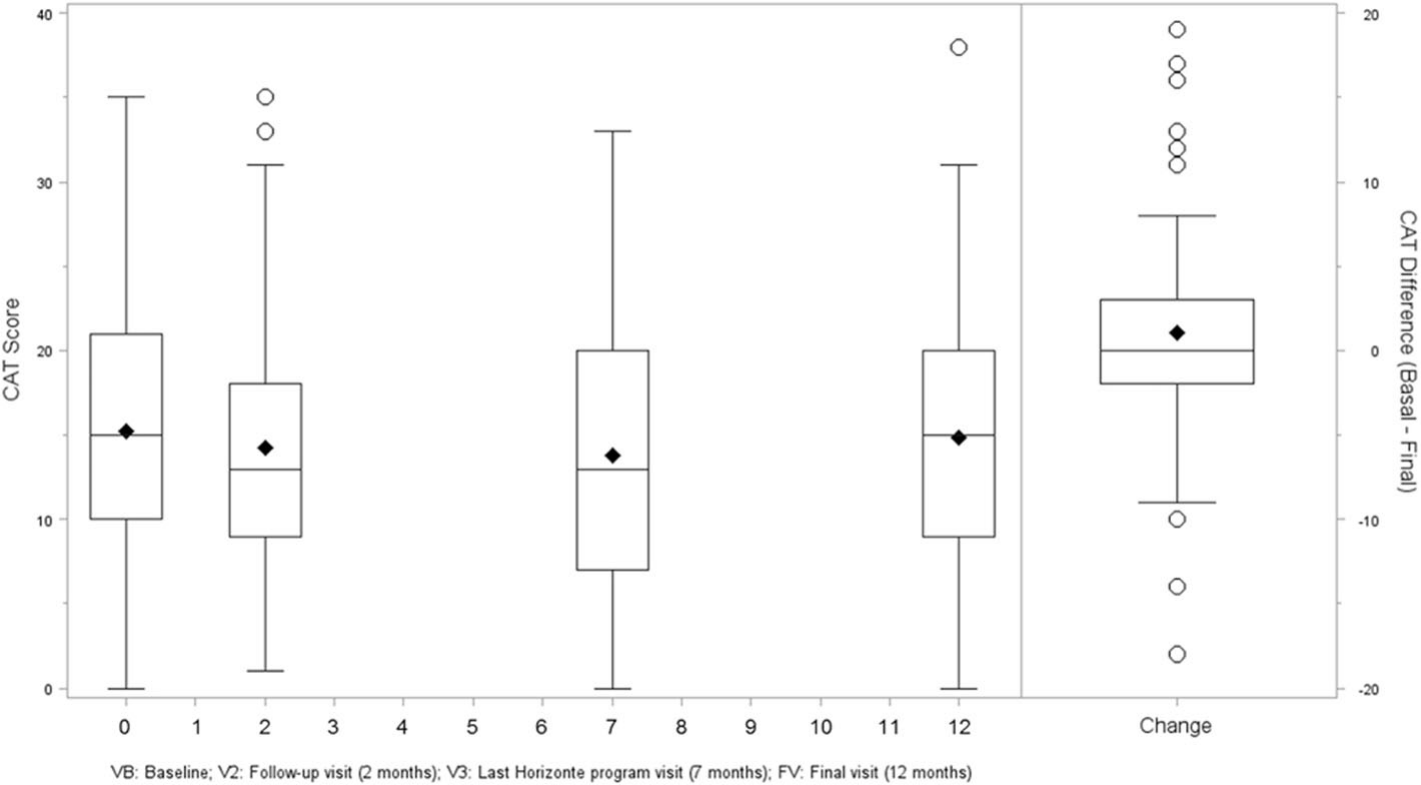
COPD assessment test (CAT) scores at baseline, at the first (V2) and second (V3) follow-up visits and at the end of the study (FV)

At baseline, patients had relatively high CAT scores: [15.3 (SD = 7.6)]. Throughout the study period, CAT scores improved an average of -0.4 (95 % CI: -1.6; 0.8) points, although not statistically significant (*p* = 0.530, paired t-Student) (Fig. 3). Worse health status measured by CAT was associated with longer duration of COPD (0.14 increase of CAT per year; *p* = 0.018), severe and very severe GOLD stages (1.9 and 3.3 points more than mild stages respectively; *p* = 0.015), higher scores in the mMRC (6.2 points more in those with mMRC > 2) and BODEx (5.4 points more in BODEx >4; *p* < 0.001), and being a current smoker (2.3 increase compared with ex-smokers; *p* = 0.026); whereas a better health status measured by CAT was associated with higher FEV1(%) (0.09 decrease per % unit; *p* < 0.001), longer walking autonomy (2.9 and 5.2 points less than <30 min walkers among those walking 30–60 min and > 60 min respectively; *p* < 0.001) and higher compliance measured by the Morisky- Green questionnaire (2.6 points less in compliant patients; *p* = 0.005).

### Significant Improvements in CAT

A total of 41.9 % (95 % CI 31.9–52.0) patients significantly improved their reported health status (CAT decrease by 2 points or more). Univariate analysis showed that significant improvement in CAT was associated with baseline CAT scores [high CAT score 19.2 (±7.5) vs. low CAT score 12.4 (±6.4); OR = 1.15, 95 % CI: 1.07–1.24; *p* < 0.001] and being non-compliant as measured with the Morisky-Green questionnaire [62.5 % (15/24) of non-compliant vs 34.7 % (24/69) of compliant patients significantly improved CAT scores; OR = 3.13, 95 % CI: 1.19–8.19; *p* = 0.021). After controlling for the effect of all variables in a multivariable logistic regression model, the only factor that remained associated with CAT change (“significant improvement” vs. “no improvement”) was the baseline CAT score (Table 4).

**Table 4:** Main objective analysis: Decrease in at least two points in CAT between the baseline and the final visit. Subgroup analysis: univariate and multivariate models

Variable	Category	Univariate analysis OR (CI 95 %)	Multivariate analysis OR (CI 95 %)
Gender	Women	--------	
	Men	0.50 (0.17–1.48)	
Age		0.97 (0.93–1.02)	
Age	≤65 years	--------	
	>65 years	0.48 (0.20–1.11)	
BMI		1.00 (0.92–1.08)	
BMI classification	Normal (18.5–24.9)	--------	
	Overweight (25.0–29.9)	1.37 (0.49–3.82)	
	Obesity (≥30.0)	0.87 (0.31–2.49)	
CAT		1.15 (1.07–1.24)	1.15 (1.07–1.24)
Time of evolution COPD (years)		0.96 (0.91–1.03)	
Previous exacerbations	2–3	--------	
	≥4	0.42 (0.11–1.65)	
FEV1(%) Postbronchodilation		1.01 (0.98–1.03)	
GOLD stage	Mild-moderate	--------	
	Severe-very severe	0.96 (0.42–2.21)	
Dyspnea	≤2	--------	
	>2	0.67 (0.27–1.63)	
BODEx index	≤4	--------	
	>4	0.88 (0.38–2.07)	
Minutes walking a day	<30	--------	
	30–60	1.48 (0.54–4.07)	
	>60	1.00 (0.36–2.78)	
Morisky-Green questionnaire	Compliant	--------	
	Noncompliant	3.13 (1.19–8.19)	
Significant cardiac comorbidity	No	--------	
	Yes	0.47 (0.17–1.36)	
Cardiovascular risk	None	--------	
	Increased	0.92 (0.28–3.102)	
	High	0.89 (0.27–2.89)	
	Very/extremely high	1.05 (0.33–3.38)	
Smoking status	Ex-smoker	--------	
	Smoker	1.98 (0.70–5.61)	

*COPD* chronic obstructive pulmonary disease, *BMI* body mass index, *CAT* COPD Assessment Test

### Change in the number of exacerbations

The average duration of COPD in the sample was 8.8 ± 6.8 years, while in the 12 months prior to the study, 85.1 % experienced 2 or 3 exacerbations and 14.9 % experienced 4 or more. During the Horizonte program (VB-V3), 45.0 % of patients experienced exacerbations [30.0 % moderate exacerbations, 24.0 % severe exacerbations (8.0 % exacerbations that led to emergency room visits, and 17.0 % hospitalization)]. The median number of exacerbations was 2 for total exacerbations (IQR 1-3) with a maximum of 8; 1 for moderate exacerbation (IQR: 1-2) with up to 6; 1 for severe exacerbation (IQR: 1-1) with up to 8; and 1 emergency exacerbation (IQR: 1-1) with a maximum of 5. After program completion, 57.0 % of patients had suffered exacerbations [35.5 % moderate exacerbations, 29.0 % severe exacerbations, (15.1 % emergency exacerbations, and 22.6 % exacerbations requiring hospitalization)).

**Fig. 4:**
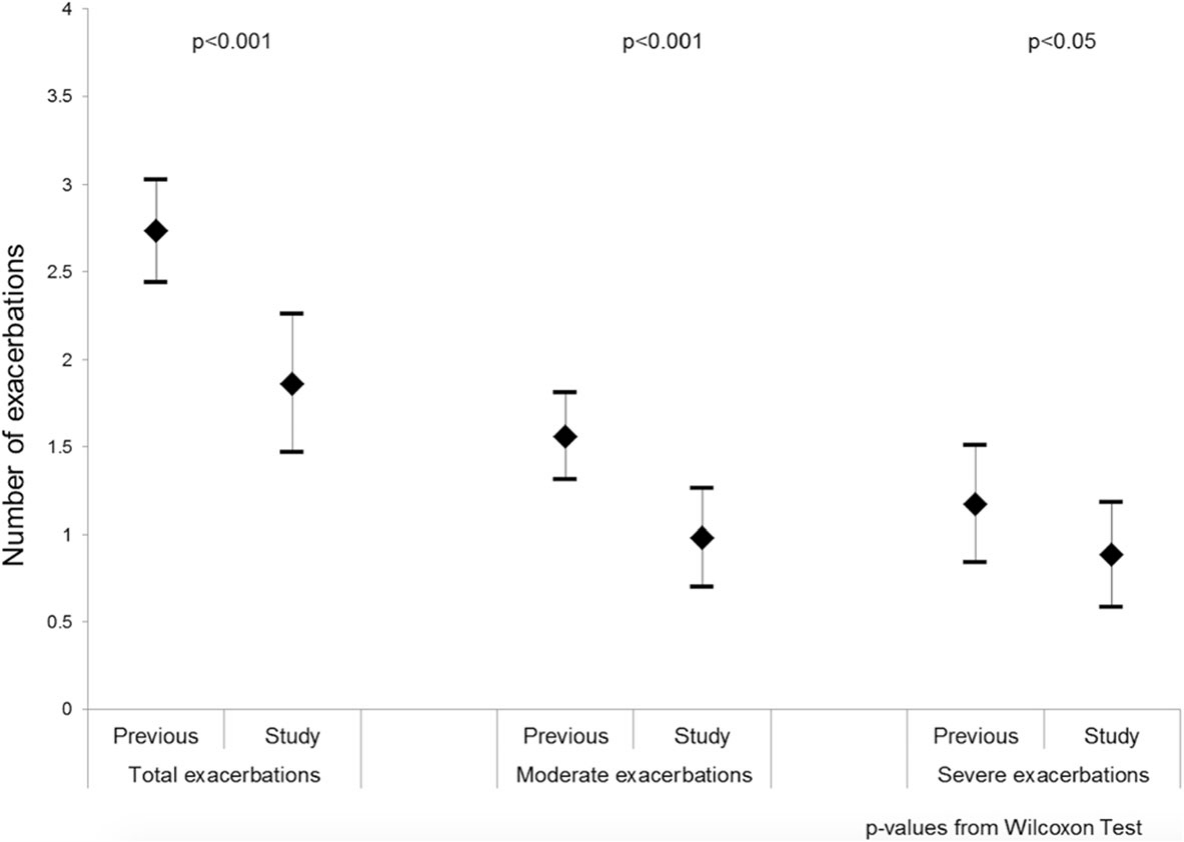
Difference in the number of exacerbation (12 months before starting the study and during the 12-months follow-up of the study)

Changes in COPD exacerbations frequency 12 months before and after the study are presented in Fig. 4. During the 12 months before the study, median total exacerbations was 2 (IQR: 2-3), while during the study it decreased to 1 (IQR: 0-3). Median change (post-pre study) in the number of exacerbations was therefore -1 (IQR: -2, 0), (*p* < 0.001). For moderate exacerbations, patients experienced a median of 2 (IQR 1-2) and, during the study, 1 (IQR 0-1). Median change (post-pre study) in the number of moderate exacerbations is -1 (IQR: -2, 0), (*p* < 0.001). Finally for severe exacerbations, patients experienced a median previous exacerbations of 1 (IQR 0-2) and, during the study, 0 (IQR 0-1), with a median change (post-pre study) in the number of severe exacerbations of 0 (IQR: -1, 0), (*p* = 0.017).

### Changes in pulmonary function

No significant differences in pulmonary function were observed from baseline. Initial mean FVE1 was 49.5 % (16.9) and at the end of the study it was 49.0 % (18.2) not statistically significant (0.0 (IQR: -5.0; 4.0), *p* = 0.822).

### Changes in adherence

Changes in adherence were of borderline significance (*p* = 0.053). According to the Morisky-Green questionnaire, at baseline 25.8 % (24/93) of patients were noncompliant. Of these patients, at the end of the study, 66.7 % (16/24) became compliant. Of the 74.2 % (69/93) of compliant patients at baseline, 91.3 % (63/69) did not change their adherence and 8.7 % (6/69) became non-compliant when the study finished.

## Discussion

The objective of the present study was to examine the impact of a remote support program on the health status of moderate/severe COPD patients. Recommendations for the use of specific support programs for patients with COPD are based on experience with other chronic diseases such as diabetes [20], coronary artery disease [21], and asthma [22]. Studies investigating the utility of such programs for COPD reported heterogeneous conclusions, and meta-analyses have argued that more studies are needed [23, 24].

Reducing the burden of disease by improving patients’ symptoms, functional status, and quality of life are important goals. There has been a substantial increase in the use of newly developed tools that measure health status and it is important for clinicians and researchers to assess these instruments’ strengths and weaknesses in providing insight into a patient’s condition and experience. Relying only on mortality and physiological outcomes could blind the clinician to the potential benefits that patients may receive from a treatment. A growing body of research utilizes end-points assessed directly by patients whose self-reported health status includes health-related quality of life and their functional status [25].

The main finding of this study is that high risk COPD patients; who received a remote support program based on disease-specific self-management principles showed an improvement in their health status in nearly half of the patients analyzed assessed with the CAT questionnaire and a significant reduction in the occurrence of exacerbations. Although we cannot identify which component of the intervention had an effect, the results nevertheless remain important, considering the limitations of current COPD treatments, the burden of the disease, and the need for effective care plans to optimize the use of limited resources.

To assess health status in COPD patients, the most widely used short questionnaires are CAT [18], the Clinical COPD Questionnaire (CCQ) [26], the Airways Questionnaire 20 (AQ20) [27], and the COPD severity score (COPDSS) [28]. In particular, CAT, AQ20, and CCQ have been assessed on their predictive value for exacerbations and mortality [29, 30]. CAT covers a broad range of effects of COPD on patients’ health including cough, phlegm, chest tightness, breathlessness going up hills/stairs, activity limitation at home, confidence leaving home, sleep, and energy. CAT has demonstrated to be the best predictive questionnaire for a series of outcomes (new ambulatory or emergency exacerbations, hospitalization, or death) in patients with moderate-to-severe COPD [31].

Some studies have shown that patients with severe COPD improve less with specific health programs compared with patients with less severe disease [32, 33]. Interestingly enough, our study suggests that non-compliant patients and COPD patients with higher CAT scores at baseline, which means a worse health status, are the patients who have benefited the most from this remote program. This could be explained, at least partially, by differences in the severity of COPD among these groups of patients which led to a better health status and by the different interventions adapted to each patient’s specific risk to non-adherence, with more intense interventions for those patients at higher risk. Duration of intervention has been demonstrated to be an important variable in a previous meta-analysis [14]. The health-related quality of life scores, COPD-related ED visits, and hospital admission rates were similar between groups during the initial 3- to 6-month follow-up. However, all of these outcomes reached statistical significance when the program outcomes were compared after a 12-month follow-up. The 7-month duration of the Horizonte program could have limited the statistical significance of some outcomes.

Exacerbations are important determinants of prognosis in patients with COPD, and are associated with health status [34, 35], lung function [36], mortality [37], and economic costs [38]. Prodromal symptoms of an exacerbation commonly occur up to a week before a discernible reduction in lung function [39], and about one-half of patients who seek treatment in an emergency department report having had characteristic symptoms for at least 4 days [40]. Early treatment of exacerbations has been shown to reduce morbidity and effect on quality of life [41] and the remote program implemented in the study seems to be very effective at reducing exacerbations, even in patients who are difficult to control and with a long duration of COPD disease. Moreover, the reduced number of exacerbation of the present study confirms previous reports [42, 43] of improvements in exacerbations with disease specific interventions. On the contrary, in the study carried out by Van Wetering et al., no reductions in the number of exacerbations were obtained [44]. This is probably associated with a less advanced COPD in the patients included in that study.

Adherence to treatment was obtained in 100 % of the sample at the end of the program and it was maintained until the end of the study, which could suggest an improvement in the compliance of the patients. Moreover, the results of the Morisky-Green questionnaire showed a tendency to an increase in adherence.

Regarding the impact of the program on smoking status, significant heterogeneity has been observed in previous studies assessing the impact of disease-specific education programs. Only one out of three trials [45–47] reported significant differences in the number of current smokers between groups following the implementation of a disease-specific education program, maybe due to the fact that COPD patients lose their motivation after being discharged from the hospital, and supervised home-based care could be more effective [48].

This approach of care through a continuum support and favouring self-management does not require specialized resources and can be easily implemented. The present study supports its use as an integral part of the long-term care of patients with moderate to advanced COPD, but further studies are needed to confirm their effectiveness.

Study limitations include lack of completion of the a priori minimum sample size and those of any observational study, like neither randomization of the sample nor reduced room for inferences. Additionally it would have been desirable to have a control group to indetify the effect of Horizonte program itself. Nevertheless, our a priori defined recruitment procedure to minimize sampling bias, systematic evaluation of consecutive COPD patients from different centres, and evaluation of patients’ health status using a standardized assessment instrument (CAT) strengthens our results. Our study characteristics are very similar to those in COPD patients with frequent exacerbations [14], so it is reasonable to assume that non-included cases would have had similar characteristics.

## Conclusions

The implementation of a remote support program for high risk COPD patients results in improvements of patients’ health status, particularly those with an initially poor health status, and it effectively reduces exacerbations.

## Additional file

Additional file 1: Description and workflow of the Horizonte Program. (DOCX 92 kb)
