# Supplementary material for: The evaluation of a remote support program on quality of life and evolution of disease in COPD patients with frequent exacerbations
Source: BMC Pulm Med. 2016 Nov 8;16:140. doi: 10.1186/s12890-016-0304-3 (PMC5100103; doi:10.1186/s12890-016-0304-3)
Supplement: Additional file 1: — Description and workflow of the Horizonte Program. (DOCX 92 kb) [file 12890_2016_304_MOESM1_ESM.docx]

**Additional file 1**

**DESCRIPTION OF THE HORIZONTE PROGRAM**

*Patient Journey 1*

Patients, enrolled on the programme, that were on new treatment less than 4 weeks before the day of enrolment. Those patients received a workbook on week 1 and the first call from a nurse one week after the enrolment, a second call was made at week 2 and the third call at week 4 after de enrolment. On the third call new patients were profiled as high or low risk based on their beliefs and symptoms.

Segment 1- Patients with new treatment sub-segment A - high risk: received by mail 5 magazines and up to 4 minimagazines based on their beliefs and symptoms. If those patients ops for emails they received 2 email/week during months 1 and 2 , and 1 email/week at months 3, 4, 5, 6 and 7. If they ops for SMS the frequency was 3 SMS/week during months 1 and 2, and and 2 SMS/week at months 3, 4, 5, 6 and 7.

Segment 1- Patients with existing treatments , sub-segment A - low risk: received by mail 2 magazines and up to 4 minimagazines based on their beliefs and symptoms. If those patients ops for emails they received 1 email/ week during months 1 and 2 and 2 email/month during months 3, 4, 5, 6 and 7. If they ops for SMS the frequency was: 1 SMS/week during months 3, 4, 5, 6 and 7.

*Patient Journey 2*

Patients, enrolled on the programme, on inhaler treatment or on oral and inhaler treatment for more than 4 weeks and patients on inhaler treatment less than 4 weeks. Those patients received a workbook on week 1 and a call from a nurse one week after the enrolment.

High Risk (Segment 2 –Patients with existing treatment, sub-segment B): For those patients a second call was made at week 2. Patients also received by mail 5 magazines and up to 4 minimagazines based on their beliefs and symptoms. If those patients opts for emails they received 2 email/week during months 1, 2 and 3 and 1 email/week at months 4, 5, 6 and 7. If they ops for SMS the frequency was 3 SMS/week during months 1, 2 and 3 and 2 SMS/week at months 4, 5, 6 and 7.

Low risk (Segment 2 – Patients with existing treatment, sub-segment A and Segment 1 - New patient, sub-segment B): Patients received by mail 2 magazines and up to 4 minimagazines based on their beliefs and symptoms. If those patients opts for emails they received 1 email /week during months 1,2 and 3 and 2 email/month during months 4, 5, 6 and 7. If they opts for SMS the frequency was: 2 SMS / week during months 1,2 and 3 and 1 SMS/week during months 4, 5, 6 and 7.
